# Liquid Biopsy: minimal-invasive analysis of somatic alterations

**DOI:** 10.1515/medgen-2023-2061

**Published:** 2023-12-05

**Authors:** Ariane Hallermayr

**Affiliations:** MGZ – Medizinisch Genetisches Zentrum München Germany

Liquid biopsy describes the non- or minimal-invasive collection of body fluids for the analysis of circulating markers including circulating tumor cells (CTCs), circulating free DNA (cfDNA) and extracellular vesicles (EVs). Liquid biopsy is very promissing for companion diagnostics (CDx) [13] and disease monitoring in cancer [12]. However, the concept is not limited to cancer but rather is used in a broad range of clinical applications, such as non-invasive prenatal testing (NIPT) [1], the diagnosis of monogenic mosaic diseases [7, 14] or patient surveillance following organ transplantation [17].

This special issue provides a great overview of the current status of a variety of circulating markers and clinical applications in the field of liquid biopsy. This emerging field is of high relevance for human genetics as in many cases DNA, the backbone of genetic diagnosis, is the underlying material anlyzed.

## The potential of different circulating markers in liquid biopsies

A variety of circulating markers can be analyzed from different body fluids. While blood is the main focus of liquid biopsy-based analysis, CTCs, cfDNA and EVs are also observed in other body fluids, including urine, cerebrospinal fluid (CSF) or saliva. CTCs are recognized to be the first known analyte in liquid biopsies, having been described as early as 1869 [2]. Initial studies mainly focused on the metastatic potential of CTCs. For disease monitoring in cancer CTC enumeration was established as a predictive marker and using single-cell analysis it is possible to characterize CTCs even functionally [10]. While CTCs are specific to cancer, cfDNA and EVs represent promissing biomarkers for a variety of diseases since they are released from all cell types. Even though the biological cargo present in EVs, represent the functional characteristics of a disease in viable cells, diffiulties in standardization for EV isolation still limit der applicability for diagnostics [19]. Therefore, in a current diagnostic setting cfDNA analysis, is the most promissing. cfDNA is easy accessible and advances in technology enable the application in a variety of clinical settings, including disease monitoring in cancer, NIPT or the genetic diagnosis of mosaic diseases [8].

## Clinical applications of liquid biopsy

The analysis of liquid biopsies is promissing in a broad range of clinical settings. Guidelines from the European Society of Medical Oncology (ESMO) recommend the detection of actionable variants in circulating tumor DNA (ctDNA) for CDx in a variety of cancers [13]. In specific instances in non-small cell lung cancer (NSCLC) and breast cancer (BC) ctDNA analysis is reimbursed by German health insurances. Further, ctDNA analysis enables the detection of minimal residual disease (MRD) and recurrence as well as treatment monitoring. Prospective studies showed promissing results for ctDNA guided decision making for or against adjuvant chemotherapy in early stage colorectal cancer (CRC) [16] and for ctDNA guided changes in treatment upon the detection of resistance markers in metastatic BC [3].

The analysis of genetic material from liquid biopsies comes with several implications in the field of human genetics. Since cfDNA is released from a variety of tissues, cfDNA analysis is not limited to cancer. The probably best-established application of liquid biopsy-based diagnostics is NIPT for the detection of fetal genetic alterations. Further, cfDNA analysis provides great potential for the identification of the underlying genetic cause of mosaic diseases.

## This special issue on liquid biopsy

Within this special issue we aimed to provide an overview of the emerging technologies and clinical applications in the field of liquid biopsy. Detailed reviews on cfDNA [5], CTCs [15] and EVs [11] describe the broad spectrum of circulating markers present in liquid biopsies and advances in technologies for the highly sensitive analysis of these markers. Further, the spectrum of clinical applications such as the early detection of cancer and MRD detection [4], treatment monitoring and molecular profiling of tumors [18], the implications of cfDNA analysis in human genetics [6] and NIPT [9] are described in detail in reviews on these topics.

Our goal was to emphasize the importance of Liquid Biopsy in the field of human genetics. The main focus of current liquid biopsy-based diagnostics is the analysis of cfDNA. This requires highly sensitive methods for which precise analytical validation and standardization are essential to enable clinical interpretation. Particular consideration is needed since many clinical applications aim to provide patients with personalized medicine. Accordingly, it is important in the field of human genetics to educate ourselves in the field of molecular medicine in order to enable high quality diagnostics.

With great sadness, I have to mention that Michael and I could not finalize this special issue together due to his sudden death. However, I would like to take this opportunity to thank him for the outstanding collaboration. Prof. Speicher was a great mentor and I had the opportunity to gain a lot of knowledge through him. He was a pioneer in the field of liquid biopsy and contributed substantially to the progress of this field. In an obituary, which is included in this issue, his achievements are described in detail.
